# Optimizing Time to Antibiotic Administration in Children with Possible Febrile Neutropenia through Quality Improvement Methodologies

**DOI:** 10.1097/pq9.0000000000000236

**Published:** 2019-11-21

**Authors:** Beech Burns, Melinda Hartenstein, Amber Lin, Denise Langley, Erin Burns, James Heilman, Mary Tanski, Linda Stork, O. John Ma

**Affiliations:** From the * Department of Emergency Medicine, Oregon Health and Science University, Portland, Ore.; † Department of Pediatric Critical Care, Oregon Health and Science University, Portland, Ore.; ‡ Department of Pediatric Hematology and Oncology, Oregon Health and Science University, Portland, Ore.

## Abstract

Febrile neutropenia is a potentially life-threatening complication of chemotherapy in pediatric oncology patients. Prompt initiation of antibiotic therapy may minimize morbidity and mortality associated with this condition, and time to antibiotic (TTA) administration <60 minutes is used as a quality benchmark by many institutions. We implemented a quality improvement initiative to achieve TTA < 60 minutes in >80% of eligible patients in the pediatric emergency department.

**Methods:**

After collecting baseline data, we employed consecutive PDSA cycles to (i) reduce time to antibiotic order after patient arrival; (ii) expedite the preparation of antibiotic by pharmacy; and (iii) enable antibiotic ordering before patient arrival. Statistical process control methodologies were used for key outcome measures to compare pre-intervention, post-intervention, and maintenance periods.

**Results:**

Comparing pre-intervention and post-intervention years, mean TTA decreased from 64 to 53 minutes and the percentage of patients receiving antibiotics in <60 minutes increased from 59% to 84%. Improvements were sustained in the maintenance period of the project, with mean TTA administration of 44 minutes and 85% of patients receiving antibiotics within our stated goal.

**Conclusion:**

Through a series of PDSA cycles, we decreased TTA and increased the percentage of febrile neutropenia patients receiving antibiotics in <60 minutes.

## INTRODUCTION

Febrile neutropenia (FN) is a major cause of morbidity and mortality in children with cancer, and empiric therapy with antimicrobials has become standard treatment for patients with FN^1^. Current research demonstrates that the longer a patient is febrile and neutropenic before empiric treatment, the greater the risk of serious infection.^2^ Furthermore, data suggest that minimizing time to antibiotic (TTA) administration may result in decreased adverse outcomes, including need for resuscitation, intensive care unit admission, and death.^3–5^

In light of these considerations, the Infectious Disease Society of America and the National Comprehensive Cancer Network recommend TTA for FN patients of 60 to 120 minutes of presentation to the emergency department (ED).^6,7^ Additionally, US News and World Report has been tracking TTA as a Quality of Care measure since 2010; in 2012, a survey of pediatric oncology centers revealed that 45% of respondents were using TTA as a quality measure, with over 90% using a standard of <30 or 60 minutes of time from presentation to administration in the outpatient setting, ED, and inpatient unit.^8^ Despite the widespread use of TTA administration as a quality measure and the potential benefit to patient safety, multiple studies demonstrate that individual hospitals are frequently not meeting this goal in immunocompromised patients.^8–10^

We began collecting data on pediatric patients presenting to our ED with FN in 2009. In 2010, the pediatric emergency medicine team collaborated with the oncology service to create a clinical pathway stipulating that patients would be administered antibiotics without waiting for absolute neutrophil count (ANC) results, with a goal TTA of <60 minutes. This process change decreased mean TTA to 59–60 minutes in 2011–2012.^10^ While this TTA was consistent with our stated goal, only 46% of total patients presenting with possible FN were receiving antibiotics in <60 minutes. Beginning in April 2015, the goal of this quality initiative was to ensure that >80% of patients were receiving empiric antibiotics within 60 minutes 1 year after project implementation. We adopted a goal of 80% as we felt this was a realistic target and represented a significant improvement from the previous baseline of 46%.

## METHODS

This study was conducted at a pediatric emergency department (PED) within a major academic medical center in the United States with an annual volume of 15,000 patients per year. Our quality improvement team included the PED nurse manager, the PED medical director, the ED quality director, the ED pharmacist lead, and a clinical informatics pharmacist. For baseline measurements, we collected data on all pediatric oncology patients who presented to the PED with a reported history of fever within the preceding 24 hours or documented fever in the PED from April 2014 to March 2015. We excluded patients who received antibiotics at another hospital and were subsequently transferred to our PED. Because this was a quality improvement initiative, it was not considered human subjects research and was exempted from IRB requirements at our institution.

Data were collected via EMR system reports and aggregated monthly to generate mean TTA administration and the overall percentage of febrile, possibly neutropenic patients receiving antibiotics within 60 minutes. TTA administration was defined as the difference between the time of patient arrival in the PED and the time that antibiotic infusion begins. To identify possible unintended consequences of emphasizing the care of potentially neutropenic febrile patients, we also examined time to bronchodilator therapy and time to corticosteroid administration in patients with asthma as balancing measures.

**Fig. 1.**
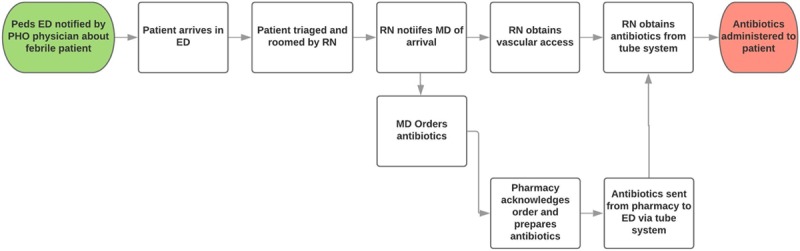
Process map for patients presenting to the ED with possible febrile neutropenia. Process map describing the ED workflow from the time the ED is notified of the incoming patient to the time of antibiotic administration. PHO, pediatric hematology oncology; RN, registered nurse; MD, medical doctor.

To identify factors contributing to delays in antibiotic administration, we employed an A3 problem solving approach and developed a process map to better understand our workflow (Fig. 1). The key steps included (1) time patient arrives to PED, (2) time antibiotic order was placed, (3) time required for antibiotic preparation by pharmacy, (4) time following preparation to arrival of the antibiotic to the ED, and (5) time to initiate antibiotic infusion once it has arrived. Vascular access, an important barrier to prompt antibiotic delivery in this population, had been addressed in previous QI efforts at our institution and was not addressed in this project. In February and March of 2015, the clinical and pharmacy teams performed multiple drills during the day and evening shifts to determine the time required for antibiotic preparation. The initial analysis determined that preparation and delivery to the PED generally required about 47 minutes.

### Interventions

#### PDSA #1 (April 2015)

The aim of our initial PDSA cycle was to minimize the interval following patient arrival to placement of the antibiotic order in the EMR. In 2010, our institution began administering cefepime empirically to all patients with possible FN, in accordance with published clinical practice guidelines recommending a broad-spectrum antimicrobial with pseudomonal coverage.^11^ Before this time, antipseudomonal antibiotics were reserved for patients with ANC < 500 only, while ceftriaxone was administered to the remainder. This QI initiative and its rationale are described separately.^10^ Because our practice is to administer cefepime regardless of ANC, this antibiotic may be ordered immediately upon patient arrival. Initial examination of the relevant intervals in this process revealed that, to ensure that there is adequate time for preparation in pharmacy and delivery in the ED in <60 minutes, antibiotics needed to be ordered consistently within 13 minutes.

We began to emphasize this goal to both nursing and physician staff members in April of 2015, introducing this new initiative at the Pediatric ED Unit-Based Nursing Practice Committee and the Pediatric Emergency Medicine Section meetings. Utilizing the quality improvement board in our ED, a visible display of metrics concerning current quality initiatives, we collected real-time information regarding causes of delay in antibiotic administration using an abnormality tracker. An abnormality tracker is a tool for documenting deviations from the established standard work for a process of interest. For example, for this initiative, if a patient failed to have antibiotics ordered within 13 minutes, this was noted by the bedside or charge nurse and reasons for potential delay were documented (eg delay in rooming patient, provider not immediately available, etc.). This tool was reviewed twice daily during nursing shift huddles facilitated by the charge nurse to ensure completion. It was collected by the PED nurse manager and the multidisciplinary team reviewed the aggregate results to identify obstacles to be addressed. Lastly, monthly emails were sent to providers globally reminding them to promptly order antibiotics for these patients and audit and feedback via email was performed with individual providers by the PED Medical Director.

Time to ordering antibiotics did decrease, from 35 minutes in April 2015 to 12 minutes in June, with corresponding decreases in TTA from 62 to 53 minutes. However, our percent of patients receiving antibiotics in <60 minutes remained generally under 80%.

#### PDSA #2 (June 2015)

The longest interval contributing to total TTA was preparation of the appropriate antibiotic by pharmacy and delivery to the ED. Several strategies to reduce the time required to prepare the antibiotic were considered, and ultimately we elected to attempt improved door to antibiotic delivery times through use of an order set. Though an order set existed, it was not always utilized by providers and the pharmacy staff did not uniformly appreciate the urgency of preparation in this population. The order set was therefore reviewed and revised, with antibiotic from the order set now prioritized as “urgent” in the EMR. The physician staff were provided education on the use and rationale of order sets and pharmacy personnel were trained to prioritize antibiotic orders from this order set against other competing medication orders. In June 2015, work to improve order set compliance was initiated. Order set usage did improve, up to 100% in July. However, though TTA administration decreased, the percentage of patients receiving antibiotic in <60 minutes remained below 80% month to month. After 2 months of encouraging order set use with suboptimal improvement, our team moved to our final PDSA cycle.

#### PDSA #3 (August 2015)

In virtually all cases, the PED receives telephone notification from the referring oncology physician that a potentially neutropenic child will be coming to the ED within 1 hour. We reasoned that if the antibiotic could be prepared at the time of notification, the interval containing the preparation of the antibiotic could shift to a period before the child’s arrival, substantially decreasing the total TTA and improving the percentage of patients meeting the <60 minute target.

Several challenges existed at this point. First, antibiotics could not be ordered in our EMR for a patient who had not yet arrived in the ED. Working with our informatics team, we were able to modify the EMR to allow the physician to enter an antibiotic order once the ED encounter chart had been created in anticipation of the patient’s arrival. Second, pharmacy could not prepare the proper quantity of antibiotic without knowing the patient’s recent weight, normally obtained at the time of arrival. After discussion with pharmacy and our pediatric oncology team, we agreed it is safe to calculate antibiotic doses based on the patient’s most recent weight, which is virtually always documented in the EMR at a clinic visit within the previous month.

Lastly, we educated our staff regarding the process of ordering antibiotics before the patient’s arrival. This was accomplished by creating a Job Breakdown Sheet that was distributed via email and by reviewing the new strategy at meetings of the ED faculty, PED section, and Unit-Based Nursing Practice Committee. The Job Breakdown Sheet was placed on the department quality board next to the abnormality tracker to reinforce the new process and the Pediatric ED Medical Director continued monthly audit and feedback with individual providers.

### Statistical Methods

Statistical process control was used to analyze mean TTA and the proportion of patients receiving antibiotics in <60 minutes across each study period, including the pre-intervention period (April 2014–March 2015), the intervention period (April 2015–March 2016), and the maintenance period (April 2016–March 2017). To analyze data related to means we employed x-bar charts and for proportional data we used p-charts. Standard rules for defining special cause variation were utilized.

## RESULTS

Included patients are described in Table 1. The total number of patients was similar in each phase of the project, while a greater percentage of patients were neutropenic in the maintenance phase. Average ED length of stay was comparable among the phases and PICU admissions were very rare in all 3 time periods.

**Table 1.**
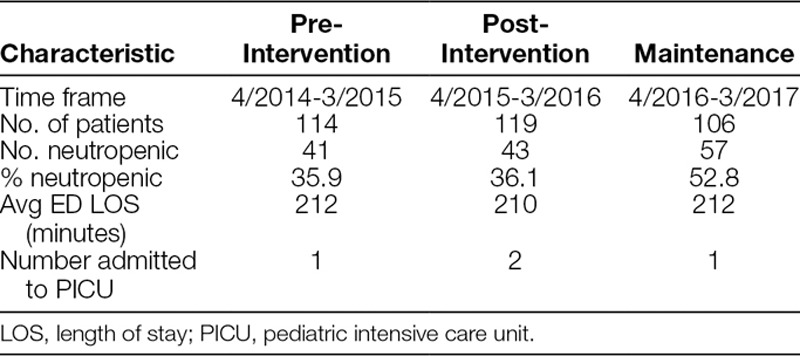
Characteristics of Included Patients

**Fig. 2.**
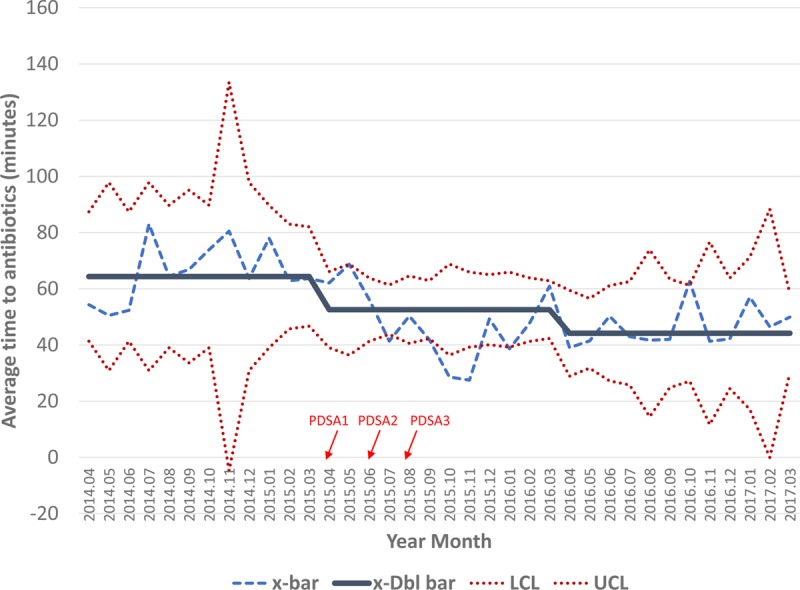
Time from patient arrival to antibiotic administration (in minutes). X-bar chart demonstrating the average TTA administration during each period and the associated control limits. The x-bar line represents the average time for each month, while the x-dbl bar represents the mean of means for the period. Special cause variation was observed between the pre-intervention and intervention periods, improvements maintained during the maintenance period. The timing of the PDSA cycles is indicated by the red arrows. Abx, antibiotics; PDSA, plan-do-study-act; x-bar, subgroup mean; x-Dbl bar, mean of means; LCL, lower control limit; UCL, upper control limit.

**Fig. 3.**
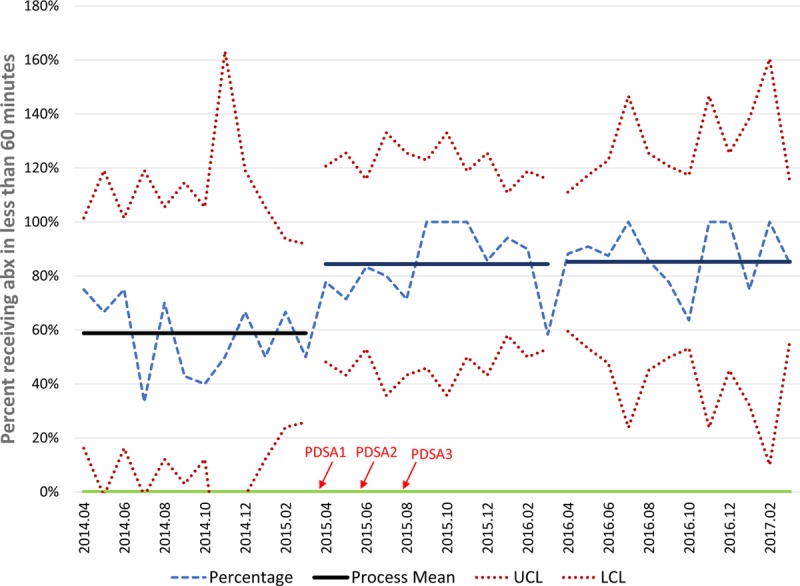
Percentage of patients meeting goal of antibiotics in <60 minutes. P-chart demonstrating the percentage of visits receiving antibiotics in <60 minutes during each period and the associated control limits. Comparing the pre-intervention and post-intervention periods, an increased percentage of patients received antibiotics in <60 minutes; improvements were sustained during the maintenance period. The timing of the PDSA cycles is indicated by the red arrows. PDSA, plan-do-study-act; LCL, lower control limit; UCL, upper control limit.

Data around mean TTA and percentage of patients meeting our goal are represented in Figures 2 and 3. Mean TTA for the preintervention period was 64 minutes, with 59% of eligible pediatric patients receiving antibiotics in <60 minutes. Mean TTA in the post-intervention period was 53 minutes, well below our target of 60 minutes and a decrease of 11 minutes from the pre-intervention period. Special cause variation was observed between the pre-intervention and intervention periods, with 8 points in a row below the centerline. Additionally, 84% of patients presenting with fever and possible neutropenia received antibiotics within 60 minutes, with special cause variation observed by way of 8 points in a row above the centerline. This result represents a 25% increase from our baseline measurement in the pre-intervention period.

PDSA cycle 1 occurred in April of 2015, while PDSA cycle 3 occurred in August of 2015. After conducting the first improvement cycle, mean TTA decreased from 62 minutes in April to 56 minutes in June, with percentage meeting goal increasing from 78% to 83%. The second improvement cycle further decreased TTA to 44 minutes in July, with 80% in the goal range. After executing the third improvement cycle, ordering antibiotics before the patient’s arrival, average TTA decreased to 42 minutes in September and the percentage meeting goal increased to 100%. While we did not continue to have this level of success, our average TTA for the post-intervention period was well under 60 minutes and >80% of patients received antibiotics within 60 minutes. Further, these gains were sustained in the maintenance period of the project, with a mean TTA of 44 minutes and with 85% of patients receiving antibiotics in <60 minutes.

### Balancing Measures

**Fig. 4.**
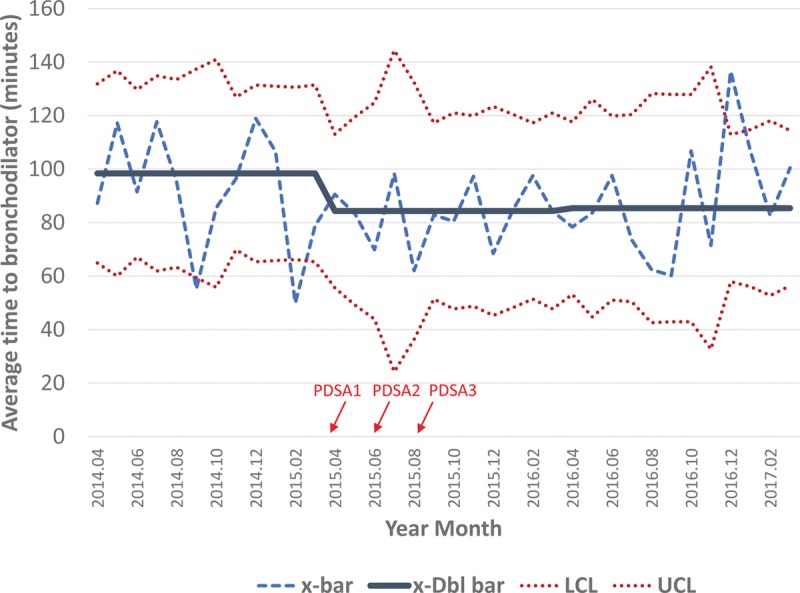
Time from patient arrival to first bronchodilator administration (in minutes). X-bar chart demonstrating average time to first bronchodilator administration during each period and associated control limits. The x-bar line represents the mean for each month, while the x-dbl bar represents the mean of means for the period. There is a decrease in mean time to bronchodilator administration between the pre-intervention and intervention periods, largely sustained during the maintenance period. The timing of the PDSA cycles are indicated by the red arrows. PDSA, plan-do-study-act; x-bar, subgroup mean; x-Dbl bar, mean of means; LCL, lower control limit; UCL, upper control limit.

**Fig. 5.**
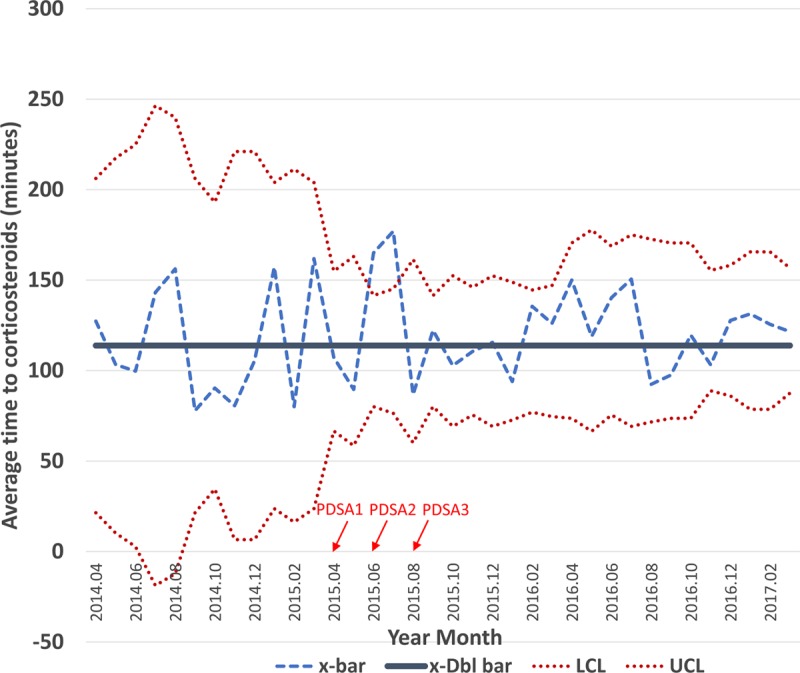
Time from patient arrival to corticosteroid administration (in minutes). X-bar chart demonstrating average time to corticosteroid administration during each period and associated control limits. The x-bar line represents the mean for each month, while the x-dbl bar represents the mean of means for the period. No sustained differences were observed between the pre-intervention, intervention, and maintenance periods. The timing of the PDSA cycles is indicated by the red arrows. PDSA, plan-do-study-act; x-bar, subgroup mean; x-Dbl bar, mean of means; LCL, lower control limit; UCL, upper control limit.

Among patients presenting to our ED with an asthma exacerbation during the interval of interest, there were no significant increases in the average time to administration of first bronchodilator or corticosteroids during the pre-intervention, post-intervention, or maintenance phases (Figs. 4 and 5). Times to initial treatment with a bronchodilator were 98, 84, and 85 minutes during the pre-intervention, post-intervention, and maintenance intervals, respectively. For corticosteroid administration, we achieved mean times of 114, 111, and 122 minutes for each interval.

## DISCUSSION

Through implementation of a series of PDSA cycles, we were able to achieve our aim of increasing the percentage of patients receiving antibiotics in <60 minutes, with average TTA times of 53 minutes and 84% of patients within our goal in the post-implementation period. These improvements were sustained during the maintenance period in the year following the effort through continued audit and feedback. Process mapping and performing drills to define the time required for various steps were instrumental in identifying targets for improvement, and a multidisciplinary team involving physicians, nurses, pharmacy, oncology, and informatics was essential in achieving success.

Results like ours have been rarely described in the ED setting. While inpatient units and outpatient clinics have had fair success in achieving target TTA goals, with 75% of respondents in a large survey of pediatric oncology centers reporting mean TTA of <60 minutes, EDs have been far less successful, with <50% reporting times <60 minutes.^8^ A recent study examining patients evaluated in the ED achieved a substantial reduction in TTA, from 154 minutes before process implementation to 95 minutes after, but still well above the frequently utilized goal standard of <60 minutes.^9^ Another study published in 2012 did achieve an average TTA of 49 minutes for children with known neutropenia presenting to the ED, with an average TTA of 81 minutes for those with possible neutropenia.^12^ However, in this latter group with possible neutropenia, 60% were ultimately found to be neutropenic, and the large majority of these patients did not receive antibiotics in <60 minutes.

To promote antimicrobial stewardship and minimize the risk of fostering resistant organisms, the ideal approach would be to administer empiric antibiotics only to patients with FN and to avoid unnecessary broad-spectrum antibiotics in patients with fever but without neutropenia. To achieve this, an institution would require mechanisms to rapidly identify patients with possible FN, obtain complete blood counts with differentials, secure prompt vascular access, and prepare broad-spectrum antibiotics at the appropriate dose with adequate safety checks. Given the multiple relevant constraints involving cost, safety, availability of personnel, and technology, this ambitious goal has proven elusive in the ED. The compromise we have accepted is administration of rapid antibiotics to all oncology patients with possible FN, understanding that some will receive an antibiotic that is not strictly necessary. We will continue to pursue improvements permitting rapid determination of ANC and explore risk stratification in the hope of adopting a more targeted approach.

Regarding the quality improvement methods employed in this study, based on existing literature, we anticipated that audit and feedback would be an effective method for monitoring and reinforcing improvement efforts.^13,14^ However, it is critical that the proposed improvement activity be perceived as reasonably attainable. We encountered understandable resistance when we asked providers to place an order for antibiotics within 13 minutes of patient arrival, with concerns of task interruption, competing clinical responsibilities, and missed or delayed communications between the physician and triage nurse on patient arrival. Despite the best efforts of providers, this target was simply difficult to meet in a sustainable way. In approaching cycle 2, we realized that quality improvement for complex processes must involve all relevant parties. Further improvement in reducing TTA and improving percentage of patients who received antibiotics in <60 minutes required examination of all parts of care delivery, and ultimately involved collaboration with our informatics team, pharmacists, ED nursing, and emergency providers. Finally, and perhaps most importantly, interventions in quality improvement projects should be designed to make workflow easier, which will earn investment from stakeholders. For providers, ordering antibiotics before patient arrival consolidated related tasks: the provider received a call directly from the referring oncologist, opened the patient chart in the EMR, documented relevant clinical information, and entered the antibiotic order at the same time. This represented a potential improvement in efficiency from the previous system, which had required the provider to open the chart upon taking the referral and then open the chart again to order antibiotics after the nurse notified the MD that the febrile patient with possible neutropenia had arrived.

Our study had a few limitations. For one, it was conducted in the PED of a single academic medical center with a strong working relationship with the oncology division. Because an expectation has been created that any fever in an oncology patient should prompt the family to call the oncology provider, notification of the impending arrival of a patient with possible FN and preordering antibiotics is made possible. In systems where the destination of care for a patient cannot be anticipated or the oncology team is not notified of an ill oncology patient, such communications cannot be made and such a system will not work. Lastly, the major delays in the TTA will differ in settings where the antibiotics are kept in the ED or are mixed by nursing staff and do not require delivery from a central pharmacy.

## CONCLUSIONS

Through a series of PDSA cycles, we successfully decreased TTA and increased the percentage of FN patients receiving antibiotics in <60 minutes to over 80% and sustained these improvements.

## DISCLOSURE

The authors have no financial interest to declare in relation to the content of this article.

## References

[1] Hann I Viscoli C Paesmans M A comparison of outcome from febrile neutropenic episodes in children compared with adults: results from four EORTC studies. International Antimicrobial Therapy Cooperative Group (IATCG) of the European Organization for Research and Treatment of Cancer (EORTC). Br J Haematol. 1997;99:580–588. 9401070 10.1046/j.1365-2141.1997.4453255.x

[2] Meckler G Lindemulder S Fever and neutropenia in pediatric patients with cancer. Emerg Med Clin North Am. 2009;27:525–544. 19646652 10.1016/j.emc.2009.04.007

[3] Fletcher M Hodgkiss H Zhang S Prompt administration of antibiotics is associated with improved outcomes in febrile neutropenia in children with cancer. Pediatr Blood Cancer. 2013;60:1299–1306. 23417978 10.1002/pbc.24485

[4] Pakakasama S Surayuthpreecha K Pandee U Clinical practice guidelines for children with cancer presenting with fever to the emergency room. Pediatr Int. 2011;53:902–905. 21418423 10.1111/j.1442-200X.2011.03363.x

[5] Salstrom JL Coughlin RL Pool K Pediatric patients who receive antibiotics for fever and neutropenia in less than 60 min have decreased intensive care needs. Pediatr Blood Cancer. 2015;62:807–815. 25663663 10.1002/pbc.25435 PMC4413050

[6] Freifeld AG Bow EJ Sepkowitz KA; Infectious Diseases Society of America. Clinical practice guideline for the use of antimicrobial agents in neutropenic patients with cancer: 2010 update by the infectious diseases society of america. Clin Infect Dis. 2011;52:e56–e93. 21258094 10.1093/cid/cir073

[7] Segal BH Freifeld AG Baden LR Prevention and treatment of cancer-related infections. J Natl Compr Canc Netw. 2008;6:122–174. 18319048 10.6004/jnccn.2008.0013

[8] McCavit TL Winick N Time-to-antibiotic administration as a quality of care measure in children with febrile neutropenia: a survey of pediatric oncology centers. Pediatr Blood Cancer. 2012;58:303–305. 21509930 10.1002/pbc.23148 PMC3150359

[9] Cash T Deloach T Graham J Standardized process used in the emergency department for pediatric oncology patients with fever and neutropenia improves time to the first dose of antibiotics. Pediatr Emerg Care. 2014;30:91–93. 24457498 10.1097/PEC.0000000000000077

[10] Lamble A Nguyen T Lindemulder S A clinical pathway to reduce time to antibiotic administration in pediatric cancer patients with fever and potential neutropenia. Journal of Clinical Pathways. 2015;1:33–42.

[11] Lehrnbecher T Robinson P Fisher B Guideline for the management of fever and neutropenia in children with cancer and hematopoietic stem-cell transplantation recipients: 2017 update. J Clin Oncol. 2017;35:2082–2094. 28459614 10.1200/JCO.2016.71.7017

[12] Volpe D Harrison S Damian F Improving timeliness of antibiotic delivery for patients with fever and suspected neutropenia in a pediatric emergency department. Pediatrics. 2012;130:e201–e210. 22711718 10.1542/peds.2012-0153

[13] Ivers N Jamtvedt G Flottorp S Audit and feedback: effects on professional practice and healthcare outcomes. Cochrane Database Syst Rev. 2012;13:CD000259. 10.1002/14651858.CD000259.pub3 PMC11338587 22696318

[14] Vratsistas-Curto A McCluskey A Schurr K Use of audit, feedback and education increased guideline implementation in a multidisciplinary stroke unit. BMJ Open Qual. 2017;6:e000212. 10.1136/bmjoq-2017-000212 PMC5699124 29450304

